# Voltammetric Determination of Flunixin on Molecularly Imprinted Polypyrrole Modified Glassy Carbon Electrode

**DOI:** 10.1155/2016/5296582

**Published:** 2016-05-08

**Authors:** Abd-Elgawad Radi, Nadia Abd El-Ghany, Tarek Wahdan

**Affiliations:** ^1^Department of Chemistry, Faculty of Science, Damietta University, Damietta 34517, Egypt; ^2^Department of Chemistry, Faculty of Science, Suez Canal University, El-Arish 45111, Egypt

## Abstract

A novel electrochemical sensing approach, based on electropolymerization of a molecularly imprinted polypyrrole (MIPpy) film onto a glassy carbon electrode (GCE) surface, was developed for the detection of flunixin (FXN). The sensing conditions and the performance of the constructed sensor were assessed by cyclic, differential pulse and (DPV) square wave voltammetry (SWV). The sensor exhibited high sensitivity, with linear responses in the range of 5.0 to 50.0 *µ*M with detection limits of 1.5 and 1.0 *µ*M for DPV and SWV, respectively. In addition, the sensor showed high selectivity towards FXN in comparison to other interferents. The sensor was successfully utilized for the direct determination of FXN in buffalo raw milk samples.

## 1. Introduction

**Scheme 1:**
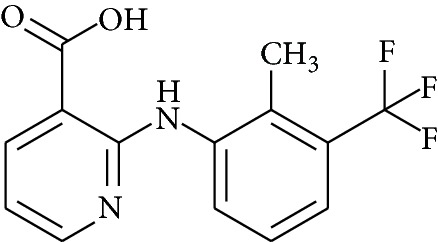
Structure of FXN.

Flunixin (2-[[2-methyl-3-(trifluoromethyl)phenyl]amino]pyridine-3-carboxylic acid) (FXN) (Scheme 1) is a nonsteroidal anti-inflammatory drug (NSAID). The major effect of FXN, as all the NSAIDs, is the nonselective reversible inhibition of both isoforms of cyclooxygenase, COX, an enzyme responsible for inflammation and pain via catalysis the formation of prostaglandins through the arachidonic acid cascade [1]. It is widely used in veterinary medicine for the treatment of various diseases in several animal species. FXN shows nonnarcotic analgesic and antipyretic properties. FXN is used to treat the musculoskeletal conditions, calf pneumonia [2–4], respiratory disorders [5], endotoxin acute mastitis, endotoxemia [6, 7], and acute coliform mastitis [8]. FXN has been shown to improve the clinical effects seen after experimentally induced coliform mastitis in the goat [9] and has a beneficial effect on the course of experimentally induced bovine pneumonic pasteurellosis (*Pasteurella haemolytica*, biotype AI) [5]. FXN, in conjunction with standard antimicrobial therapy, may be of potential value in the treatment of the infection with* P. haemolytica*, common infection in sheep flocks [10].

The widespread use of the drug in food-producing animals can lead to residues of potential risk for the consumers of food. The potential adverse effects from residues include gastrointestinal erosion and ulceration, renal vasoconstriction and insufficiency, skin reaction, headache, and central nervous system depression [11]. The dosing formulation of FXN was the second most frequent violative residue detected by the USDA-Food Safety Inspection Service (FSIS) in 2010 [12]. To protect consumers from health-threatening residues, several analytical methods have been used for analyzing FXN in biological matrices, such as voltammetry [13], high performance liquid chromatography [14–16], thin-layer chromatography [17], gas chromatography [18–22], and capillary electrophoresis [23]. The analytical methods for the determination of FXN in milk included high-performance liquid chromatography (HPLC) [24], liquid chromatography/mass spectrometry (LC/MS) [21, 25, 26], and gas chromatography/mass spectrometry (GC/MS) [21]. However, these methods have some disadvantages such as consumption of large amounts of organic solvents, long analytical procedure, and expensive equipment.

Electroanalytical methods are gaining increasing attention in the drugs analysis for their high sensitivity and fast analysis time [27]. Molecular imprinting polymers (MIPs) and the design of mimetic receptor system with predetermined recognition sites for target molecule have rapidly been developed. MIPs have several advantages over their biological counterparts, including low cost, ease of preparation, and good physical and chemical stability in a wide range of experimental conditions and many solvents. The methods of preparation of MIPs include soft lithography, molecular self-assembly, and electropolymerization. The electropolymerization methods have several attractive features such as the easy adherence of the polymeric films to the surface of conducting electrodes of any shape and size and the ability to control the thickness of the films under different depositions conditions. Electropolymerization has been successfully utilized for the preparation of electroactive and electroinactive polymers on a variety of conductive surfaces. The MIPs are prepared by copolymerization of functional and cross-linking monomers in the presence of a template through covalent or noncovalent interactions; the subsequent removal of the template creates cavities complementary in size, shape, and orientation of their binding sites to those of the template, which could selectively rebind the analyte. The MIPs are able to selectively recognize analytes ranging within biologically relevant markers, drugs, and agrochemicals. Until now, MIPs are widely used in several fields, such as chromatographic separation, solid phase extraction (SPE), drug release, reaction catalysts, enzyme mimics, and sensors. Polypyrrole (Ppy), the first routinely electrochemically synthesized polymer, has received a great attention due to its convenience of preparation, high stability, and wide range of applications. Ppy is well known to be partially cross-linked with no need to use a cross-linking comonomer; it can be easily electropolymerized on various substrate materials. Ppy shows an electrochemical redox activity, which allows the entrapment of a wide range of compounds.

The objective of the present study was to develop a selective, sensitive, and fast analytical method for trace level detection of FXN using GCE modified with electropolymerized molecularly imprinted Ppy film and DPV and SWV as detection techniques. A MIPpy film was prepared by in situ electropolymerization of Py in the presence of FXN onto the GCE surface and FXN was incorporated during the electropolymerization of Py in aqueous solution using CV into the imprinted Ppy film on the electrode surface. There was no need for sample preparation and time consuming extraction steps other than centrifugation for the determination of FXN in the milk.

## 2. Experimental

### 2.1. Materials and Reagents

Pure FXN was kindly supplied by Delta Pharma, Egypt. Its purity was found to be 99.9% as stated by the supplier and used as received. The stock solution of FXN (1.0 mM) was prepared by dissolving in 5.0 mL of methanol and then diluting with water to 25.0 mL. The working solutions were prepared by diluting the stock solution with 0.20 M phosphate buffer solution. Py (Sigma-Aldrich) was of reagent grade quality and was used as received. The preparation of the aqueous solutions was carried out using ultrapure water.

### 2.2. Electrochemical Measurements

All electrochemical measurements were performed with Bio-Logic SAS Electrochemical Analyzer, Model SP50, controlled by EC-Lab Express Version 5.52 software (Bio-Logic SAS, France). A conventional three-electrode cell, incorporating a working glassy carbon electrode (BAS model MF-2012, *ϕ* = 3 mm), a saturated Ag/AgCl reference electrode (BAS model MF-2063), and a platinum wire counterelectrode (BAS model MW-1032), was employed. The electrodes were fitted to a glass cell to hold 10 mL of the electrolyte.

### 2.3. Procedures

The GCE was polished to a mirrorlike finish successively with 0.1 *µ*m and 0.05 *µ*m alumina slurries on microcloth pads (Buehler, Lake Bluff, IL, USA). The electrode was sonicated in ultrapure water to remove trace alumina from the surface (Deltasonic Meaux, France). The GCE was immersed in 0.1 M NaClO_4_ supporting electrolyte including 10 mM Py and 1 mM FXN. The imprinting procedure was achieved by CV in a potential range between −0.55 V and +1.40 V at a scan rate of 100 mV s^−1^. The extraction of FXN from the imprinted polymer was obtained electrochemically by cycling between −0.55 and 1.30 V in 0.2 M phosphate buffer pH 4.7, for ten cycles until all FXN molecules were stripped from the imprinted Ppy film. A control experiment, nonimprinted polymer modified electrode (NIP) was fabricated under the same experimental conditions without adding FXN. For CV, DPV, and SWV measurements, the initial and final potential were variable, depending on the pH value and the cut-off of the electrolyte.

The procedure of the milk samples is described as follows. The buffalo raw milk samples (2 mL) from farm animals not treated with FXN were spiked at 5.0 to 15.0 *µ*g/mL. The mixture was first vortexed for 5 min; 2 mL acetonitrile was added, vortexed for 1 min, and centrifuged at 4500 rpm for 10 min, and the supernatant was collected. A 1.0 mL aliquot of the supernatant was diluted to 10 mL with 0.2 M phosphate buffer pH 4.7 in a volumetric flask. The solution was finally transferred into the voltammetric cell for analysis without further pretreatment and analyzed directly by voltammetry.

## 3. Results and Discussion

### 3.1. Electrochemical Oxidative Behaviors of FXN on the Bare GCE

**Figure 1:**
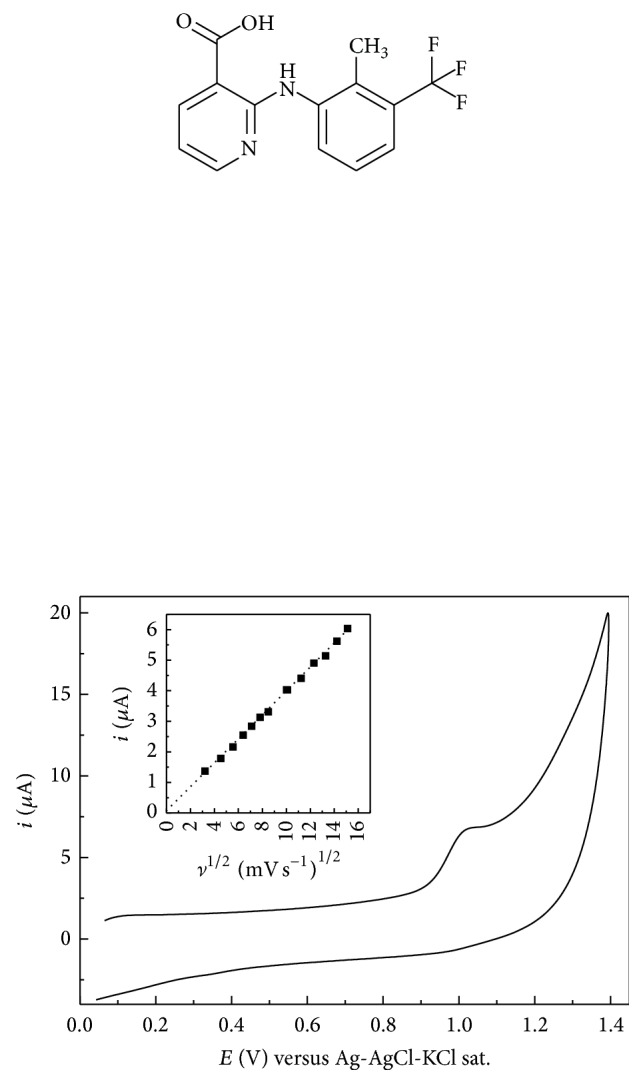
Cyclic voltammograms of 5.0 × 10^−5^ M FXN on GCE in 0.2 M phosphate buffer solution (pH 4.7). Scan rate 50 mV s^−1^. Inset: plot of anodic peak current (*i*_*p*_) for the oxidation of FXN against the square root of potential sweep rate (*ν* ^1/2^).

The cyclic voltammogram recorded for 5.0 × 10^−5^ M FXN in the phosphate buffer solution pH 4.7 at the bare GCE is shown in Figure 1. FXN yielded a single oxidation peak in the anodic scan with no reduction peak occurring on the backward scan, indicating that the oxidation was irreversible. The effect of potential scan rate (*ν*) on the peak potential (*E*_*p*_) and peak current (*i*_*p*_) of FXN was evaluated. Cyclic voltammetry revealed the absence of reverse wave/peak at any scan rate. There was an increase of peak current when the scan rate was increased. The oxidation peak current (*i*_*p*_) was plotted as a function of the square root of the scan rate (*ν* ^1/2^), and the resulting plot (*i*_*p*_/*ν* ^1/2^) is presented in Figure 1 inset. *i*_*p*_ is directly proportional to *ν* ^1/2^, indicating that the reaction is under diffusion control. This was also confirmed by plotting the logarithm of *i*_*p*_ as a function of the logarithm of *ν*, which yielded a straight line with a slope of 0.46 (correlation coefficient 0.998). The slopes of 1.0 and 0.5 are expected for ideal reactions of surface and solution species, respectively [28]. The peak potential (*E*_*p*_) was also dependent on *ν*. *E*_*p*_ shifted to more positive potentials on increasing *ν*, which confirms the irreversibility of the oxidation process. Thus, the value of *α*_*a*_ *n* (where *α*_*a*_ is the charge transfer coefficient and *n* is the number of electrons transferred in the electrooxidation of FXN) can be calculated from the slope of *E*_*p*_ versus ln⁡*ν*. The slope *b* = (2.303/*RT*)/*α*_*a*_ *nF* was 0.026, taking *R* = 8.314 J mol^−1^ K^−1^, *T* = 298 K, and *F* = 96,480 C mol^−1^ to be 1.03. In general, *α*_*a*_ is assumed to be 0.5 for totally irreversible electrode process [29]. Accordingly, the number of electrons (*n*) transferred in the electrooxidation of FXN was considered to be ~2 electrons.

**Figure 2:**
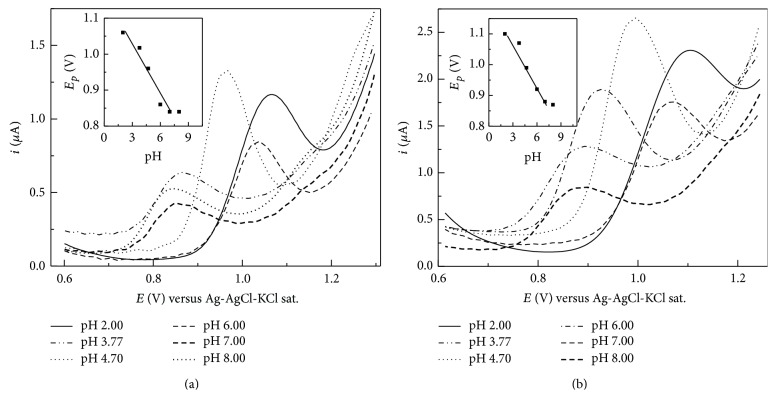
(a) Differential pulse and (b) square wave voltammetry and of 1.0 × 10^−5^ M FXN in 0.2 M phosphate buffer solutions at different pH on GCE. Insets: *E*_*p*_ versus pH plots. DPV conditions were pulse amplitude, 50 mV; pulse width, 50 ms; and scan rate, 20 mV s^−1^ and SWV conditions were pulse amplitude, 25 mV; frequency, 15 Hz; and potential step, 4 mV.

In all cases, the pH value plays an important role in the electrochemical behavior of organic analytes.  Figure 2 shows the voltammetric oxidation of FXN on the GCE in phosphate buffer solutions in the pH range 2.0–8.0 using DPV and SWV. The highest anodic peak current was obtained in the range of pH 4.7. In addition, It was found that the redox peak potentials shifted to more negative values as pH increased, and the oxidation peak potentials present a linear relationship with the pH values: *E*_p_ (V) = 1.18–0.048 pH (*r* = 0.991); *E*_p_ (V) = 1.21–0.047 pH for DPV and SWV, respectively (insets of Figure 2). The number of protons transferred (*m*) was then obtained from the slope of the relationship of *E*_*p*_-pH according to the equation *m* = (*dE*_*p*_/dpH)(*α*_*a*_ *n*)/0.059. These results suggest that one proton was involved in the rate-determining step of the oxidation process of FXN.

### 3.2. Electrochemical Imprinting of FXN into Ppy

**Figure 3:**
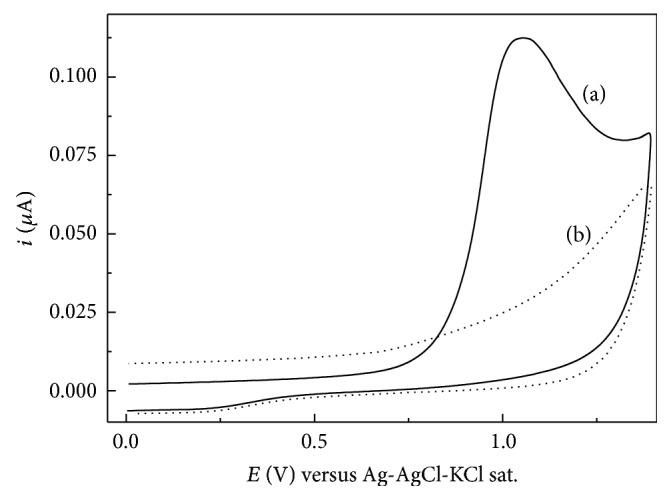
Cyclic voltammograms of (a) MIPpy/GCE sensor before removal of template and (b) MIPpy/GCE in 0.2 M phosphate buffer solution (pH 4.7). *ν* = 50 mV s^−1^.

**Figure 4:**
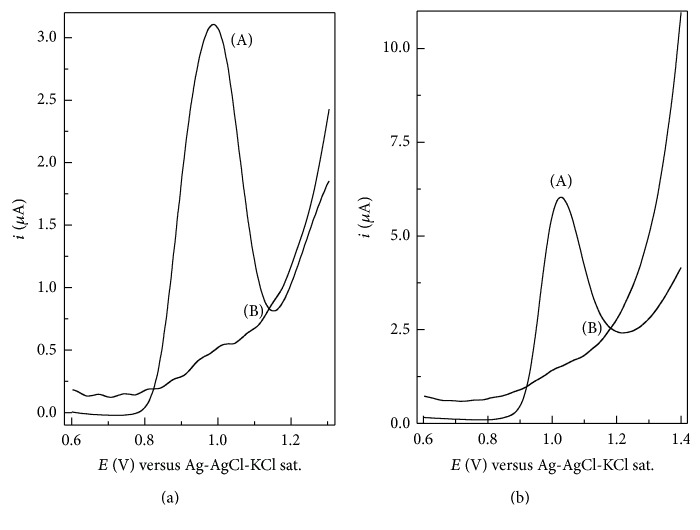
(a) Differential pulse and (b) square wave voltammograms of 5.0 × 10^−5^ M FXN in phosphate buffer solution (0.2 M, pH 4.7) at (A) MIPpy and (B) MIPpy/GCE. Other conditions are shown in Figure 2.

The formation of MIPpy on the GCE surface was evaluated using CV.  Figure 3 shows the CV responses of the MIPpy/GCE electrode before the removal of FXN template; a typical current response of FXN with an oxidation peak at about 1.05 V was clearly observed, which can be attributed to the electrochemical oxidation of FXN, whereas no peak could be observed for the MIPpy/GCE. Since the CV measurement was performed in a FXN-free solution, it was implied that the CV response was due to the oxidation of FXN embedded in the polymer matrix during the electropolymerization process of Py. The removal of template will create recognition sites complementary to the molecular shape, size, and functionality of FXN within the Ppy film which would selectively rebind FXN in solution. The rebinding affinity of the MIPpy was evaluated using DPV and SWV.  Figure 4 shows the DPV and SWV responses of MIPpy/GCE and MIPpy/GCE in the rebinding experiments after incubating in PBS (pH 4.7) containing 5.0 × 10^−5^ M FXN. For MIPpy/GCE, a significant oxidation peak has appeared, whereas no peak was observed for the MIPpy/GCE. The significant current response of imprinted electrode can be related to the presence of recognition cavities in the MIPpy film. The template molecules can enter into the cavities successfully due to the similarity in size and shape to the cavities. However, recognition cavities towards template molecules are absent in the MIPpy/GCE.

The number of accessible recognition sites in the MIPpy film is found to be dependent upon the cycle number, monomer concentration, and template concentration, which will in turn affect the electrochemical response of the MIPpy sensor. The current response of the MIPpy/GCE to FXN depends on the thickness of MIPpy film on the GCE surface, which can be controlled by the number of cycles during the electropolymerization. The current response increased with the cycle number up to 5 and then decreased with further increase of cycle number. The low current response at less cycle number is probably due to fewer recognition sites formed in the MIPpy film. However, more cycles than needed can cause the formation of excessive thickness of MIPpy film resulting in less accessible imprinted sites situated in the inner area of the MIPpy film which cannot be easily removed from polymer matrix. Therefore, the optimal electropolymerization cycles were chosen to be 5. The effect of monomer concentration on the analytical response of imprinting and nonimprinting GCE to FXN was also studied. The increase of the monomer concentration may cause a rapid polymerization and enhance the sensor sensitivity. However, a high concentration of the monomer might cause a nonselective electrochemical response to the template [30]. The optimal monomer concentration was chosen to be 10 mM for the next experiments. The electropolymerization of Py has been investigated at different concentrations of FXN template from 0.5 to 15.0 mM. The lower concentration resulted in a decreased current response as the amount of template molecules was too little and created fewer numbers of recognition sites in the MIPpy film. The binding capacity of MIPpy increased continuously with template concentration and reached saturation at 1.5 mM, which was chosen as the optimal template concentration for the electropolymerization.

The pH of the solution is one of the most important factors influencing the rebinding process. The effect of pH on rebinding of FXN on the MIPpy from aqueous solutions was investigated with an initial concentration of 5.0 × 10^−5^ M at different pH values (2.0–8.0). The best response was observed for pH 4.7. In acidic pH range the COOH group of FXN is mainly in the neutral form, while the MIP molecule exists in ionic form by the protonation of NH_2_ to NH_3_^+^ at the electrode-solution interface. This phenomenon favors the formation of hydrogen bonds between carboxyl group of the FXN and protonated amine and facilitates the access of the FXN molecules into their specific cavities. At a high pH (pH > 7.0), a decrease of binding capacity of FXN on the polymer was noted, probably due to the fact that at pH > 7.0, the amine groups from polymer tend to exist in neutral form which results in interactions with the MIPpy of lower strength compared to the electrostatic interactions between protonated amine and carboxyl groups. This study suggests that pH tuning can be used to further improve the performance of MIPs.

The incubation time is another important parameter affecting the rebinding process. Under the optimized conditions, the adsorption dynamics experiment was carried out by recording the current responses of MIPpy/GCE, which was immersed in 5.0 × 10^−5^ M FXN in phosphate buffer (pH 4.7) for different time from 1 min to 30 min using DPV and SWV techniques. The results indicated that the MIPpy/GCE possessed a fast response time within almost 5 min, which could be ascribed to the better site accessibility and lower mass-transfer resistance of thin MIPpy film on electrode surface.

The DPV and SWV parameters including pulse height, frequency, pulse time, scan increment, and scan rate are interrelated and have a combined effect on the voltammetric response. The effects of DPV and SWV parameters on the current response of MIPpy/GCE for 5.0 × 10^−5^ M FXN in phosphate buffer (pH 4.7) after accumulation time of 5 min was made. The optimized parameters were the ones that did not deform the peak potential or increased peak width. The various parameters were optimized with a once-at-a-time strategy. For DPV, the optimized parameters were pulse height *P*_*H*_ = 2.5 mV (studied range = 1–50 mV), pulse width *P*_*W*_ = 50 ms (studied range = 5–500 ms), step height *S*_*H*_ = 10 mV (studied range = 1–50 mV), and step time *S*_*T*_ = 500 ms (studied range = 50–1000 ms). The scan rate is directly given by *S*_*H*_/(0.001*S*_*T*_) to give an optimized scan rate of 20 mV s^−1^. For SWV, the variation of pulse amplitude in the range of 5 to 500 mV was associated with an increased oxidation peak and broadening of the peak. The magnitude of oxidation signal was constant with the interval time in the range from 5 to 500 ms and the background current declined with increasing interval time. Next, the frequency in the 10–100 Hz range at constant amplitude of 25 mV and step potential of 4 mV was evaluated. The oxidation peak was increasing and above the value of 15 Hz; no reproducible signal was obtained for the frequency higher than this value. The SWV optimal conditions were pulse amplitude, 25 mV; frequency, 15 Hz; and potential step, 4 mV.

The antibiotics and analgesics may be concurrently administered in veterinary medicine for fighting infection, counteracting inflammation, and reducing fever. Here, with respect to possible interference in the detection of FXN, ketoprofen, penicillin, oxytetracycline, phenylbutazone, enrofloxacin, dexamethasone, and erythromycin were tested as coexisting species. The selectivity of MIPpy/GCE sensor was evaluated by measuring the DPV or SWV current response of 25.0 *µ*M FXN in phosphate buffer (pH 4.7) after addition of varying concentration of each interferent (25.0–250 *µ*M). In all cases, there were negligible changes in current response of FXN in the presence of tenfold excess of each interferent, as the MIPpy/GCE sensor can recognize the FXN molecules by means of shape selection and the size of functional groups. The low current response for interferents can be ascribed to their considerable difference in structure and functional groups compared to FXN, causing the fact that the cavities formed in the imprinting process cannot bind these interferents as tightly as FXN. The effect of common interfering species such as Na^+^, NH_4_^+^, NO_3_^−^, Cl^−^, CO_3_^2−^, and K^+^ and some amino acids such as phenylalanine, cysteine and tryptophan, uric acid, and ascorbic acid was also investigated by the addition of the interfering species to a solution containing 25.0 *µ*M FXN in phosphate buffer (pH 4.7). The results showed that the studied species do not interfere in the determination of FXN up to a 100-fold excess (signal change < 5%). These results demonstrated that the proposed method has a good selectivity for determination of FXN.

The reproducibility of the proposed method was evaluated by the DPV current response for 25.0 *µ*M FXN solution. The consecutive measurements were conducted by using the same electrode. The change in peak current was negligible during the first 10 measurement cycles. Thereafter, a decrease of 4.0% of the peak current was observed after using of the MIPpy for 25 times, where it is still stable and reusable, indicating that the MIPpy electrode could be used for multiple measurements. The long-term stability of the proposed sensor was also examined. The MIPpy film was stable after storage at room temperature, without significant variance as the obtained results indicated that the peak current decreased by 5.1% of the initial current after storage for one month. The fabrication reproducibility was also evaluated with six different MIPpy/GCEs by the same procedure. The RSD was 5.5% for the peak current of 25.0 *µ*M FXN which demonstrated the reliability of the fabrication.

**Figure 5:**
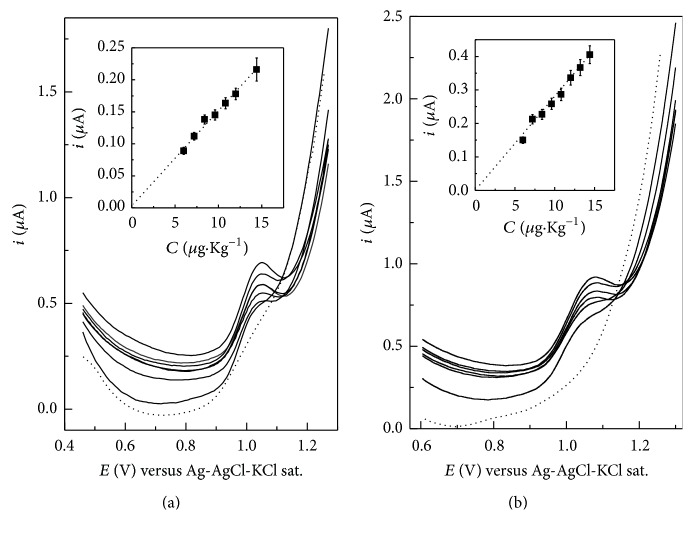
(a) Differential pulse and (b) square wave voltammograms at MIPpy/GCE sensor for different concentrations of FXN in milk in phosphate buffer solution (0.2 M, pH 4.7), dotted line: blank milk sample. Insets: calibration curves. Other conditions are shown in Figure 2.

Under the optimal conditions, the linear range for the detection of FXN using the MIPpy/GCE sensor was found between 5.0 and 50.0 *µ*M with detection limits of 1.5 and 1.0 *µ*M and high sensitivities of 0.01 and 0.02 *µ*A *µ*M^−1^ using DPV and SWV techniques, respectively. The limit of detection (LOD) of the procedure was calculated according to the 3SD/*m* criteria, where SD is the standard deviation of the intercept and *m* is the slope of the calibration curves [31]. To evaluate the reliability of the constructed sensor in real applications, the proposed sensor was used to determine FXN in buffalo raw milk samples. Typical DP and SW voltammograms, showing successive enhancement of peak current with increasing FXN concentration, are shown in Figure 5. The limit of detection was 0.15 *µ*g/Kg and 0.25 *µ*g/Kg for FXN using SWV and DPV, respectively. The recovery and the RSD of the method were always >96% and in the range 1.7–3.6%, respectively, with intraday values < 5.0% and interday values < 6.5%. There was no significant difference between slopes of calibration curves and standard addition curves. These results show that there was no interference from matrix components.

## 4. Conclusion

In summary, the present method may represent a sensitive, rapid, and cost effective alternative to that achieved by coupling the electrochemical detection to the molecularly imprinted solid phase extraction to avoid the possible interferences prior to DPV and SWV [13]. One of the simplest approaches is one-step electropolymerization of MIP film attached to electrode surface. The electropolymerization has the advantages of good adherence of the polymeric film, controllable film thickness, and creation of a direct communication between the recognition element and the surface of the transducer in a simple way. The direct DPV and SWV techniques using MIPpy/GCE sensor were successfully applied to the determination of FXN in milk samples without the necessity of sample pretreatment or any time-consuming extraction and evaporation steps prior to the analysis.
